# Annotations

**Published:** 1892-07-02

**Authors:** 


### Annotations.

Nurses and Long Skirts.

The persistent preference of a certain class of hospital nurses for long skirts is causing a good deal of surprise in quarters which do not often show a minute interest in the details of hospital life and work. *Truth* quotes the following from a nurse's letter, which appeared in the *British Medical Journal*: "After all the elaborate and costly efforts for asepticism in hospital wards, it seems to me quite inconsistent for nurses to bring into the wards the sweepings of the streets, with all the many germs they may contain. It is a dirty practice for any woman, and much more so for a nurse." *Truth* goes on to say: "I recently published a catalogue of five minutes' street-sweeping by one draggle-tailed lady in Piccadilly, and I understand that it has created some sensation in fashionable cirles. How would the sensation be intensified if it were known that the industrious street sweeper was a hospital nurse." It is to be feared that nurses are degenerating in some important respects. Time was, and not so very long ago, when the twenty thousand nurses of our British Isles were the most sensible of any class of women of equal numbers. Their experience of hospital life and hospital duties rapidly cured them of many of the little vanities and affectations so beloved of the sex, and made them rational, sober, and practical. Can it be possible that many nurses are anxious to throw away the real distinction which their unique calling confers upon them, and to assume the poor and silly frivolities of the inexperienced and undisciplined women of the lower middle-class? In the importance of the services she is capable of rendering to her fellow-creatures, the nurse is second to no woman, and indeed to no man either. But in order to make these services really great and noble, the nurse must devote herself heart and soul to her calling. If good nursing be high service to mankind, poor and perfunctory nursing is one of the meanest and most despicable of pretences. The good nurse wins love on all hands; the worthless nurse is most properly everywhere despised and detested. The least that a nurse can do is to cultivate a behaviour and daily habits which are consistent with her calling. Nothing can be more inconsistent than the wearing of clothing which must of necessity carry poison of various kinds into the ward. To nurse patients one hour, and to carry poison to them the next, is about as wicked and senseless a piece of inconsistency as can well be imagined. Surely, a single hint from such a widely-read and sharp-tongued paper as *Truth* should be sufficient to put an end for ever to the absurd practice which the article so justly condemns.

Who of us is fit to Practice?

Sir James Paget is reported to have said the other day that a surgeon on the day he receives his licence is declared fit to practice; but that, so rapid is the growth of medical science, he may be quite unfit ten years later, unless in the meantime he have kept up the habit of study. In twenty years he must be hopelessly unfit unless he have been constantly a student. This may be accepted as literally true without any rhetorical exaggeration. It has great significance for doctors; and, we venture to think, quite as much, or even more significance for the public. It is the almost universal custom for medical men to keep up their reading and study, at any rate for some years after they have received their licence to practice. But as middle life advances, unless they are fortunate enough to be in some department of special or consulting practice, the difficulties of persevering in the habit of daily reading are almost insuperable. These difficulties are not created by the doctors, but by the public. A doctor, like another man, will marry; and when he marries and has four or five children running about, they must be fed, clothed, and educated. In order to feed, clothe, and educate them the doctor must have a considerable income; and in many places the fees paid by the public are so small that not one doctor in half-a-dozen can do any more than make the requisite number of visits to secure a moderate income. By the time he has done this, he has quite exhausted his strength and vivacity, and he is compelled to rest. Reading and study under such circumstances are not only distasteful but impossible. The doctor becomes quite conscious that he is behind the times; and as a result he allows an unhappy timidity to take possession of him, a timidity which not only unfits him for practice, but renders his life dull and miserable to the last degree. But whilst the doctor and his family suffer, the public, though they are not conscious of it, suffer a great deal more, and that is quite as it should be. Every family doctor has from two to five hundred families under his care. The difference to these hundreds of people between being well doctored and ill doctored is immense. The health of many is permanently enfeebled, which need not be, if their cases were better understood, and many die who need not die. It is not the fault of the doctors; they do what they can, and no man can do more. It is the fault of the patients, who, because competition is keen among medical men, cut their doctors' fees down to the last farthing. Our sympathies are with the doctors, not with the public. Can a starving horse draw a load? Can a half-fed soldier fight victoriously through a long campaign? Neither can a doctor who has to toil like a slave, and to be harrassed worse than a slave, keep up the habit of bright and successful study. If the public desire the enormous advantage of the best science of the times in their own homes, they will do well to give a little more consideration to the question of the family doctor's fees.

Tennis or Golf for Ladies?

The *Daily Graphic*, which is an authority on games as well as on many other things, seems inclined to think that for ladies lawn tennis may go out and golf may possibly come in. It is quite desirable that golf should come in, but very undesirable that tennis should go out. Ladies have profited immensely by the out-door life which has been so fashionable for the past twenty years. The girls and younger matrons of the period are quite magnificent women compared with the namby-pamby and sickly specimens which used to be common thirty years ago. All this is due to outdoor life and exercise. It would be a pity if tennis were to share the fate of croquet, and go out, merely that golf might come in. It would mean that nine-tenths of the young ladies who now take abundant exercise would discontinue their outdoor life. Tennis can be played on a lawn of a single rood; golf requires "links" of many acres of open and little-used ground. The tennis lawn lies at the foot of every verandah. The golf links are often ten miles off, and impose a half-hour's journey by train. The stronger and more muscular young ladies should take to golf by all means wherever they have the chance. But even they will play golf all the better if they occasionally indulge in the milder pleasures of tennis. As for those who cannot afford the time for a ten-mile journey to the golf links, they will do well to assiduously cultivate tennis, at any rate until some happier game shall be discovered which can be played on the paternal lawn.

